# Reflections from the COVID‐19 pandemic on inequalities and patient and public involvement and engagement (PPIE) in social care, health and public health research

**DOI:** 10.1111/hex.13278

**Published:** 2021-08-09

**Authors:** Michael Clark, Esther van Vliet, Michelle Collins

**Affiliations:** ^1^ London School of Economics & Political Science London UK; ^2^ Nuffield Department of Primary Care Health Sciences University of Oxford Oxford UK; ^3^ Division of Health Research Lancaster University Lancaster UK

## Abstract

Patient and public involvement and engagement (PPIE) has evolved to become widely established practice in social care, health and public health research in the UK. The COVID‐19 pandemic has caused rapid change in practice in PPIE, notably in moving from face‐to‐face meetings to virtual ones. This has opened a space for reflecting on established PPIE practice, but there is a risk this is conducted too narrowly, such as only weighing our preferences and the relative pros and cons with regard to in‐person versus virtual meetings. The pandemic has also demonstrated the wide inequalities in society, and hence, we argue that an inequalities lens ought to guide a deeper and wider reflection on PPIE practice. We do not seek to criticize practice pre‐ or during the pandemic, but to encourage using the inequalities lens as a means of encouraging debate and focusing energy on a more rigorous review of PPIE practice to widen involvement in social care, health and public health research.

## 1 INTRODUCTION

Patient and public involvement and engagement (PPIE)^1^ is widely promoted in health, public health and social care research, and most major research funders in the UK require some level of inclusion of patients and members of the public in research processes. As identified in Going the Extra Mile,^1^ there is a significant risk that unless inclusion in public involvement is addressed, inequalities in health and wealth outcomes will worsen. This widening of involvement needs to occur in all research stages, from prioritization of topics, through conceptualizing projects, commissioning processes, conducting studies and reporting and seeking impact from them. The COVID‐19 pandemic has shone a light on the issue of inequalities in health and care, and we argue there is an opportunity, even necessity, to use this experience for a fundamental review of PPIE activities across health, public health and social care research.

Many public contributors fit a standard profile of 60+, white, well‐educated and with adequate finances for (initial) out‐of‐pocket costs. Additionally, many researchers tend to work with the same public contributors across projects. They are important contributors to the national endeavour of research, and long‐standing collaborations are vital in maintaining this. However, there is a danger that in resting on established ways we are too narrowly limiting our scope for opening up opportunities and addressing inequalities.

In considering inequalities, attention to the nine protected characteristics (including age, sex and race) according to the UK’s Equality Act 2010 (https://www.gov.uk/guidance/equality‐act‐2010‐guidance) is clearly needed and would be an important starting point. However, we argue the pandemic has demonstrated the need for wholesale analysis of approaches to PPIE to develop a wider and deeper understanding of who is excluded and how. This should include groups potentially excluded from PPIE practice because of their social, economic or cultural experiences and circumstances, their health status, their roles as carers, because of geographical location and/or because of infrastructure issues such as transport and/or access to technology. Their exclusion means losing important voices with potentially valuable contributions to make to improving health, public health and social care systems.

There is a need to consider how PPIE practices may be contributing to exclusion to wider public involvement. These may include an academic approach to discussing issues, formalized agendas for and styles of meetings, and locations of meetings. Some consider such settings and environments as estranging and uninviting. This set‐up also requires the public contributor to travel, and though expenses are covered, this could cause a considerable level of inconvenience for the public contributors who are less mobile due to health or care responsibilities or who live rurally. Travel expenses are often reimbursed retrospectively, which can cause financial barriers if people are required to fund expensive tickets initially out of pocket.

There have been wonderful case studies working on reducing inequalities in involvement in research, such as the NIHR INVOLVE and Research Design Services funded ‘Reaching Out’ projects (https://rds‐eoe.nihr.ac.uk/public‐involvement/nihr‐reaching‐out‐project/), PPIE grants supported by the NIHR School for Primary Care Research (https://www.spcr.nihr.ac.uk/news/spcr‐presents‐ppi‐public‐engagement‐awards) and examples in research studies from the three NIHR Research Schools. In addition, various resources have been published to support researchers in addressing inequality in their PPI work.^2^ Lessons from these can form a strong platform for developing better evidence‐based approaches to addressing inequalities in PPIE, but a more systemic review and approach to improvement is required.

In terms of addressing inequalities in involvement in other aspects of the research system, such as commissioning research, it is likely that experience is more underdeveloped, or at least less well documented. We are, for example, aware through involvement in James Lind Alliance Priority Setting Partnerships (on Social Work and Occupational Therapy priorities) of significant effort in those to widen involvement, but the lessons have so far not been codified. Similarly, commissioners of research, to our knowledge, have so far not been very systematic in reporting their experiences of widening access.

The COVID‐19 pandemic bought a profound break in established working arrangements and forced some developments in organizing PPIE that may help address inequalities, notably moving most PPIE practice to the virtual world as face‐to‐face meetings were cancelled and online platforms become the norm for holding them. This may mean that people previously excluded on the basis of having difficultly travelling to meetings or problems of access to or from shortage of facilities in buildings have been able to now participate in meetings. However, this move can also cause its own difficulties with regard to inclusion (e.g. digital exclusion if access to technology is limited) and does not address all of the issues (e.g. widening initial contact with communities to become involved, the formal nature of meetings and the fact that they are usually organized during usual office hours which may not suit everyone). In reflecting on the impact of the pandemic on PPIE and inequalities, we should not focus on the relative strengths and limitations of face‐to‐face versus virtual forms of meeting but use this as a starting point for a wholesale rethink of approaches to involving specific groups of people currently excluded.

Wider use of virtual approaches to PPIE can show other options to improve equality and address some of the issues identified above. These include using digital means to provide more flexibility on timing and means of expressing voice in PPIE. This includes online copies of documents available to comment on at times suitable to individuals. Recording online meetings and events may also have a place (with consideration of other issues such as confidentiality). There are also well‐established tools that can support accessibility, such as live captioning and translation during meetings. However, these need to be considered with the other issues touched on above, such as power dynamics, language and structure of involvement activities. Consideration of who would be excluded by using digital means would of course be an overriding need.

The COVID‐19 pandemic has provided a huge push to reflect on the methods of involvement with groups that were often not involved before. Some examples we draw from our experience are:

- Carers—Carers are central to social care and much of health care, yet they are often absent from research and research prioritization that directly affects them and to which they could make a profound contribution. Being a carer for someone at home can be extremely demanding and may not allow for travel to meetings. The move during the pandemic to hold meetings online may have provided some carers with a welcomed opportunity to contribute to research. Further creativity with virtual approaches might allow more flexibility for carers, enabling them to become more involved with research, but again we need to be mindful of the risk of exclusion from digital only approaches (as with the other groups discussed below).
- Patients with mobility‐impairing conditions—Some people can have difficulties travelling to in‐person meetings or with a lack of, for example, suitable toilet facilities at venues (such as a need for higher specification, Changing Places toilets). In this case, virtual meetings could be an enabling technology to facilitate their involvement.
- People with communication or cognitive difficulties—Some people face communication difficulties that would make it more difficult to take an active part in discussion groups. Virtual tools can open up additional communication channels in addition to talking, such as typing or drawing responses. However, it is important to keep in mind that others with communication difficulties might struggle more during an online meeting, for example due to a lack of non‐verbal communication cues. Virtual environments might at the moment not be best for those who need help with more profound communication difficulties. Talking mats, for example, have been used most extensively in face‐to‐face settings, but there may be scope to evaluate them for online communication and PPIE work as well.
- People living in locations that are geographically marginalized—There are concerns that some communities, such as people living in disadvantaged coastal areas, are not as fully involved in research as metropolitan ones. If they continue to be removed from how research systems are planned, how strategies are defined, resources allocated and priorities set, their concerns may be continually overlooked. As long as a good Internet connection is available, digital means of involvement may address some of the challenge here, but there would still remain the question of how to engage people in these communities in the first place to draw them in to online processes. Third‐sector community groups, for example, may be helpfully involved to facilitate initial, and potentially also on‐going, contact.
- People from other specific communities—This includes consideration of people from Black, Asian and Minority Ethnic and from LGBTQI + communities. Many of these communities and support groups and individuals within them will have become more familiar with using virtual environments for meeting and this can be a useful base for building more dialogue with them about how best to improve PPIE from their perspectives. Engagement can be promoted via community groups’ newsletters and social media. However, as for previous bullet points, there remains a question of how people first become interested in and aware of opportunities for involvement. Going to and more actively reaching out to communities should be considered.

While it is clear that in each of the circumstances discussed above, virtual working potentially opens up opportunities, it also leaves some questions open, and can often come with its own drawbacks with regard to improving inequalities, such as people not having access to stable Internet or devices to join virtually, fatigue of virtual meetings which may be greater for some of the groups we need to engage more, and the loss of personal connections experienced in face‐to‐face meetings. It is unlikely, then, that one method, virtual or other, will address the challenges of more equal opportunity for and voice in PPIE. We need to consider what mix of approaches are needed to build initial and on‐going engagement, and a real voice during PPIE based on evidence of who is excluded, how and what would work to redress the situation.

Superficial reflection on face‐to‐face/virtual methods of involvement will miss key points, namely that often people in marginalized groups do not see the advantages of research (or may have research fatigue when they feel there has been no change following previous research) nor of them becoming more involved in research. Addressing these points will be crucial to improving inequalities in involvement.

We do not propose to answer all the questions raised from an inequalities perspective about how to do PPIE, nor that PPIE practice before or during the pandemic was better or worse. Rather, we aim to stimulate a fundamental debate about the future of PPIE in health, public health and social care research. There is a risk that when the opportunity arises, the research community defaults to what we feel is a better form of involvement on the basis of personal preferences, such as preferring the personal interaction of face‐to‐face. A superficial examination of PPIE approaches and the experiences of the pandemic will most likely lead to PPIE being undertaken with the same people to much the same ends, upholding the current inequalities. We believe that by bringing a lens of inequalities to this process of reflecting on PPIE pre‐ and during pandemic, we can identify a better pattern of improvement to the future of PPIE. It would be essential in this process to talk to those for whom a specific approach leads to inequalities.

Though inequalities are not a new PPIE agenda item, the pandemic has given urgency to move it to the forefront of considerations about improving PPIE practice. COVID‐19 has impacted far more those in minority and lower socioeconomic groups. Equal involvement opportunities for all in social care, health and public health research are a vital means of improving the evidence to address these wide and deep inequalities in society. We want to encourage researchers to not see their PPIE work as a ‘pre‐’ and ‘post‐’ COVID‐19 approach, but as a spectrum of opportunities of which one can choose hybrid inclusive approaches that will benefit public contributors, widen access and ultimately improve the relevance of research.

## DISCLAIMER

The authors are employed for at least part of their work through the three National Institute for Health Research (NIHR) Research Schools (for social care, primary care and public health research) and draw on experiences from this work to inform this paper. The views expressed though are those of the authors and they do not necessarily reflect those of the three research schools nor of the NIHR nor the Department of Health and Social Care.

## Data Availability

Data sharing not applicable to this article as no datasets were generated or analysed during the work reported.

## Footnotes

^1^
We recognize that ‘patient’ and ‘public’ do not always fit with the research contexts we are discussing. Social care and some areas of health care, for example, prefer ‘service user’ to ‘patient’. Social care places more explicit emphasis on carers that is conveyed by the more generic ‘public’. In public health, the concept of ‘public’ can mean many different groups of people. We will, however, use the terms patient and public as the phrase PPIE is now so widely used that it is a helpful one to quickly convey our intentions and we ask readers to understand that we also include other groups of people under this umbrella.

^2^

https://www.invo.org.uk/wp‐content/uploads/2020/03/INVOLVE_RO_report_FINAL_180220.pdf

https://learningforinvolvement.org.uk/wp‐content/uploads/gravity_forms/6‐341881053f9095fe395a1f986cd7085c/2019/01/BAME_Toolkit_0.7_BME.pdf

https://emahsn.org.uk/our‐work/patient‐and‐public‐involvement/useful‐documents‐and‐links/191‐useful‐documents‐and‐links

https://www.invo.org.uk/wp‐content/uploads/2019/10/Involving‐children‐and‐young‐people‐as‐advisors‐in‐research.pdf
